# Pathological mechanisms of glial cell activation and neurodegenerative and neuropsychiatric disorders caused by *Toxoplasma gondii* infection

**DOI:** 10.3389/fmicb.2024.1512233

**Published:** 2024-12-11

**Authors:** Zihan Yang, Jiating Chen, Chi Zhang, Hongjuan Peng

**Affiliations:** Department of Pathogen Biology, Guangdong Provincial Key Laboratory of Tropical Diseases Research, School of Public Health, Key Laboratory of Infectious Diseases Research in South China (Southern Medical University), Ministry of Education, Southern Medical University, Guangzhou, Guangdong, China

**Keywords:** *Toxoplasma gondii*, glial cell activation, neuropsychiatric disorders, blood–brain barrier, neuronal damage

## Abstract

*Toxoplasma gondii* is an intracellular opportunistic parasite that exists in a latent form within the human central nervous system (CNS), even in immune-competent hosts. During acute infection, *T. gondii* traverses the blood–brain barrier (BBB). In the subsequent chronic infection phase, the infiltration of immune cells into the brain, driven by *T. gondii* infection and the formation of parasitic cysts, leads to persistent activation and proliferation of astrocytes and microglia. This process results in neuronal damages that are fatal in some cases. Through inducing systemic immune responses, *T. gondii* infection can dramatically alter the behavior of rodents and increase the risk of various neuropsychiatric disorders in humans. In this review, we explore some recent research progress on the major events involved in BBB disruption, glial cell activation and neuronal damage following *T. gondii* infection in hosts. It further discusses potential pathological mechanisms and the feasible treatment approaches for the neurodegenerative and neuropsychiatric disorders caused by *T. gondii* infection to extend our understanding for pathogenesis and preventive control of toxoplasmosis in humans.

## 1 Background

*Toxoplasma gondii* is an intracellular opportunistic protozoan that infects approximately one-third of the world population (Elmore et al., 2010). After infection by oral ingestion of protozoan cysts, *T. gondii* can transverse the blood–brain barrier (BBB), blood-eye barrier, and placental barrier, leading to widespread tissue colonization (Ferguson and Hutchison, 1987). *Toxoplasma gondii* infection in immune competent population is mostly asymptomatic, the rapidly replicated tachyzoites slowly transform to slowly replicated bradyzoites under the host’s immune pressure, existing in the form of cysts. In most organs, cysts are gradually cleared over time, but they can persist in the CNS, skeletal muscle and retina (Ferguson and Hutchison, 1987). Conversely, *T. gondii* infection in immuno-compremised patients can cause toxoplasmic encephalitis (TE), retinochoroiditis and other serious diseases, even death (Elsheikha et al., 2021). The primary infection in pregnant women can cause serious damage to the fetus, leading to abortion, stillbirth, neonatal toxoplasmosis and so on (Li et al., 2014). In immunocompromised patients, the cysts can be reactivated and transform from bradyzoites into tachyzoites, resulting in life-threatening diseases.

Acute *T. gondii* infection is usually accompanied by a strong inflammatory response in the brain, characterized by the production of a variety of proinflammatory cytokines and inflammatory mediators, such as IFN-γ and IL-6 (Suzuki et al., 2011). After entering cerebral microvessels through blood circulation, *T. gondii* invades and ruptures vascular endothelial cells (ECs) after proliferation, damaging the integrity of the BBB (Mahmoudvand et al., 2016). The EC inflammatory response caused by *T. gondii* infection activates the cytokine network, leading to the activation and proliferation of astrocytes and microglia/blood-derived macrophages (Mahmoudvand et al., 2016). Driven by cytokines, perivascular monocytes and meningeal focal monocytes infiltrate to eliminate pathogens but also cause pathological changes in the brain, which become severe in a time-dependent manner (Atmaca et al., 2014). Microglia play a role in resisting pathogen infection by releasing the proinflammatory cytokines like IFN-γ and IL-1α and recruiting immune cells from the bloodstream (Waltl et al., 2018; Batista et al., 2020). Activated astrocytes can recruit macrophages and T cells to the brain, which is considered one of the important features of TE (Wilson and Hunter, 2004).

In addition to the robust immune response elicited by the acute infection, the long-term colonization of *T. gondii* cysts in the CNS has also been implicated in a series of behavioral changes and neuropsychiatric disorders (Tyebji et al., 2019). *Toxoplasma gondii* infection has been reported to cause neurological and behavioral abnormalities in humans, cats, and mice (Vyas et al., 2007; Berdoy et al., 2000; Hermes et al., 2008). The neurotropism for *T. gondii* leads to neuronal damage and glial cell-dependent inflammatory/immune responses, which are believed to play a significant role in the development of these neuropsychiatric symptoms (Fabiani et al., 2015). However, the specific mechanisms that underlie brain injury caused by *T. gondii* infection remain elusive, yet they are critical for devising treatments and interventions for the neuropsychiatric diseases associated with the infection. This review synthesizes the existing literature to elucidate the mechanisms by which *T. gondii* breaches the BBB, alters the cerebral microenvironment, and potentially leads to a spectrum of neuropsychiatric disorders.

## 2 *Toxoplasma gondii* infection leads to notable damage of the blood–brain barrier (BBB)

After oral ingestion, *T. gondii* (bradyzoite, tachyzoite, or sporozoite) invades small intestinal villous epithelial cells, differentiates into tachyzoites, and proliferates rapidly. The parasites then enter the bloodstream from the alimentary system, infect various immune cells, and disseminate to distal organs via the mesenteric lymph nodes or blood circulation, ultimately reaching and invading the microvascular endothelial cells (ECs) of multiple organs, including the brain. Tachyzoites then enter the brain parenchyma by crossing the BBB, which is composed an EC layer, pericytes, and astrocyte end-feet (Courret et al., 2006; Dellacasa-Lindberg et al., 2011; Olivera et al., 2021). *Toxoplasma gondii* can cross the BBB through several ways: paracellular pathways by disrupting tight junctions between ECs, transcellular pathways by infecting and rupturing ECs, and Trojan horse migration by infecting peripheral-blood leukocytes (Mendez and Koshy, 2017).

The infection results in the upregulation of intercellular adhesion molecule-1 (ICAM-1) and vascular adhesion molecule-1 (VCAM-1) on ECs, along with integrins β1/β2, chemokines, and intracellular signaling pathway proteins, including Toll-like receptors (TLRs). These changes in EC membrane molecules eventually lead to the disruption of tight junctions, increased cerebral microvascular permeability, and enhanced translocation of *T. gondii* from the cortical capillaries to the brain parenchyma (Table 1).

**Table 1:** *T. gondii* infection regulates the secretion of chemokines, the expression of cell adhesion molecules, extracellular matrix and tight junction in BBB.

	*T. gondii* strain	Cell line/mouse species	Host proteins involved in the process of *T. gondii* crossing the BBB	Conclusions	References
Cytokines and cytokine receptors	RH	HFF, Hela	IL-8, CXCL1, MCP-1	Promote inflammatory infiltration of lymphocytes and monocytes in peripheral blood circulation to control the *T. gondii*.	Strack et al. (2002)
	ME49	BALB/c brain	CXCL9, CCL5	Promote the migration of effector macrophages to the brain to control the *T. gondii*.	Khan et al. (2000) and Still et al. (2020)
	76 K	C57BL/6	CXCL10	CXCL10 is necessary for CD8+ T cells to be recruited into the CNS, and the inhibition of CXCL10 will lead to increased parasite burden in CNS.	Brenier-Pinchart et al. (2001)
	ME49	C57BL/6 GFAPcre mice	ST2, MCP-1, CCL2, CXCL10	IL-33 is expressed by astrocytes during *T. gondii* infection, and its signaling acts on astrocytes via the ST2 receptor, leading to the production of inflammatory chemokines such as CCL2 and CXCL10.	Benevides et al. (2008)
	(Not mentioned)	CD8α subset of dendritic cells	CCR5, MIP-1α, MIP-1β, IL-12	Defective expression of CCR5 may lead to dysrecruitment of CCL5, Th1 cells, MIP-1α and MIP-1β.	Wen et al. (2010) and Ross et al. (2022)
	ME49	C57BL/6 CCR2-deficient mice	CCR2	CCR2-deficient mice have an increased susceptibility to ME49.	Yang et al. (2019)
	ME49	BALB/c brain	CXCL9, CXCL10, CCL5	The combined expression of these three chemokines may contribute to the migration of macrophages to the brain to control the *T. gondii*.	Ross et al. (2021)
Cell adhesion molecules and intergrins	RH, ME49, PRU, and mutant lines (*Δ*MYR1, *Δ*TgWIP, *Δ*GRA15)	Dendritic cells, bEnd.3	ICAM-1	*T. gondii* infection promotes integrin-CAM-dependent movement of DCs to ECs.	Cook et al. (2018), Seipel et al. (2010) and Barragan et al. (2005)
	(Not mentioned)	(Not mentioned)	VCAM-1, Integrin α4β1	IFN-γ induces the expression of ECs VCAM-1, which in turn binds to integrin α4β1 on CD8+ T cells, facilitating the recruitment of T cells into the brain.	Harris et al. (2007)
	RH, PRU	THP-1	Intergrin β1	*T. gondii* infection disrupt integrin β1 signal and its localization on the cell membrane, thereby altering the migratory characteristics of parasitic leukocytes.	Harris et al. (2007)
	RH	Raw 264.7	Integrin αvβ3, Integrin α2β1	Serve as the basis for *T. gondii* to cross the BBB, regulate the binding of integrin α2β1 with fibronectin and the conversion of pre-αv to mature αv subunit.	Mason et al. (2004)
ECM	Not mentioned	Not mentioned	MMP, TIMP-1	*T. gondii* infection hinders the activation of the NF-κB signaling pathway in astrocytes, which in turn activates MMPs and TIMP-1.	Da Gama et al. (2004), Clark et al. (2011)
	PRU	C57BL/6 brain	MMP-8, MMP-10	*T. gondii* infection cause T cells and CNS resident astrocytes to up-regulate the levels of MMP-8 and MMP-10.	Masocha and Kristensson (2012)
TJP	RH	Caco2, bEnd.3 cells	FAK	*T. gondii* infection can inactivate FAK function, thereby transiently disrupting the stability of intercellular TJP and promoting its intercellular migration.	Olivera et al. (2021), Huang et al. (2019) and Suzuki et al. (2005)
	GT1, ME49	CD-1 mice brain	C1q	*T. gondii* infection leads to an elevation in the levels of brain C1q, which helps clear *T. gondii* from the CNS but also results in neurodegeneration by degrading neuron connections and synapse loss.	Gazzinelli et al. (1992)
	PRU	Sprague Dawley rats and Kunming (KM) mice brain	C3	*T. gondii* infection upregulates C3, which disrupts TJ in the CNS.	Nagineni et al. (1996)

BBB, blood–brain barrier; CNS, central nervous system; MIP-1α, Macrophage inflammatory proteins-1α; MIP-1β, Macrophage inflammatory proteins-1β; DCs, Dendritic cells; VCAM-1, Vascular adhesion molecule-1; TIMPs, Tissue metalloproteinase inhibitors; TJP, Tight junction protein; C1q, Complement 1q; C3, Component 3; TJ, Tight junctions.

Activation of vascular ECs and microglia is also observed during chronic infection, resulting in persistent brain microvascular changes associated with endothelial dysfunction and continuous leukocyte-endothelial interactions (Hermes et al., 2008; Deckert-Schlüter et al., 1999; Wang et al., 2007). The persistence of *T. gondii* cysts in the CNS may continuously stimulate the production of cytokines and chemokines in the brain (Table 1), which may recruit inflammatory cells to produce neuroinflammation and lead to the entry of inflammatory mediators into the brain (Castaño Barrios et al., 2021).

### 2.1 Regulation of chemokine secretion and chemokine receptor expression

*In vitro* experiments show that the infection of human cervical carcinoma epithelial cells (HeLa) and human preputial fibroblasts (HFFs) with the *T. gondii* RH strain induces the expression and secretion of the proinflammatory chemokine IL-8 (CXCL8), growth-related carcinogens *α* (GROα/CXCL1) and MCP-1 (CCL2) (Denney et al., 1999). These chemokines are required for the recruitment of lymphocytes and monocytes to the CNS. Similarly, an *in vivo* study demonstrates that CNS ECs are important producers of proinflammatory chemokines, including CCL2, CCL5/RANTES, and CXCL10/CRG-2/IP-10, and these chemokines are key in promoting the migration of effector macrophages to the brain to combat the parasitic invasion (Daneman and Prat, 2015).

In addition to ECs, *T. gondii* infection induces the production of chemokines CXCL9/MuMIG in microglia, which promote the activation and chemotactic activity of T cells, monocytes, and macrophages (Ochiai et al., 2015). CXCL10 is also expressed by astrocytes in response to the infection (Strack et al., 2002). CXCL10 is a chemokine vital for the recruitment of CD8+ T cells to the CNS, and the inhibition of CXCL10 leads to an increased parasitic burden in the CNS (Khan et al., 2000). A recent study shows that IL-33 signaling targets astrocytes via the ST2 receptor, leading to the production of inflammatory chemokines such as CCL2 and CXCL10 (Still et al., 2020).

CCR5, a high-affinity receptor for CCL5, provides a primary signal for CD8α subsets of dendritic cells (DCs) to induce IL-12 synthesis to establish IFN-γ-dependent *T. gondii* resistance (Aliberti et al., 2000). Defective expression of CCR5 may disrupt the recruitment of CCL5, Th1 cells and macrophage inflammatory proteins 1α and 1β (MIP-1α/CCL3 and MIP-1β/CCL4) (Brenier-Pinchart et al., 2001). Similarly, CCR2 is essential for the activation of bactericidal mediators needed to control *T. gondii* replication in the CNS: CCR2-deficient mice have an increased susceptibility to ME49, while parasite replication in the CNS is not inhibited (Benevides et al., 2008). During chronic infection of *T. gondii* ME49 strain, CXCL9, CXCL10 and CCL5 are expressed in the brains of mice (Wen et al., 2010).

### 2.2 Regulation of the expression of adhesion molecules and integrins

Peripheral blood monocytes and DCs migrate to the site of inflammation in response to cytochemokines. They undergo roll, attach, and trans-endothelial migrate (TEM) under the guidance of various adhesion molecules on the surface of ECs. *T. gondii* infection induces ICAM-1 expression in DCs and ECs, and thereby enhances TEM frequency (Ross et al., 2022; Yang et al., 2019).

In addition, *T. gondii* infection promotes integrin-CAM-dependent movement of DCs to ECs (Ross et al., 2021). Previous studies have shown that IFN-γ induces the expression of VCAM-1 on ECs, which in turn binds to integrin α4β1 on CD8+ T cells, facilitating the recruitment of T cells into the brain (Wang et al., 2007). *Toxoplasma gondii* can disrupt integrin β1 signaling and its localization on the cell membrane, thereby altering the migratory behavior of parasitic leukocytes (Cook et al., 2018). Another study suggests that the expression of integrin αvβ3 by Raw 264.7 cells serves as the basis for *T. gondii* crossing the BBB. This integrin is able to regulate the binding of integrin α2β1 with fibronectin and the conversion of preαv to the mature αv subunit, a process that involves the membrane type 1 matrix metalloproteinase (MT1-MMP) signaling pathway (Seipel et al., 2010). In contrast, the level of MT1-MMP is elevated on the surface of macrophages infected by *T. gondii*. MT1-MMP promotes the degradation of EC extracellular matrix (ECM) and thus promote the TEM of the infected macrophages (Seipel et al., 2010). Therefore, this pathway may be the preferred way for the processing of the integrin prosubunit in *T. gondii*-infected macrophages (Seipel et al., 2010). In addition, human ICAM-1 can interact with parasite adhesin MIC2 on the surface of *T. gondii* and directly facilitates *T. gondii* adhesion (Barragan et al., 2005).

### 2.3 Degradation of extracellular matrix

The integrity of the ECM another important component of the BBB, also plays a crucial role in preventing invasion by pathogens. During *T. gondii* infection, MMPs degrade the ECM and promote the infiltration of T cells into the brain. However, this process can be partially inhibited by tissue metalloproteinase inhibitors (TIMPs) (Suzuki et al., 2011). The regulation of MMP activity and TIMP-1 induction in astrocytes is mediated by the NF-κB signaling pathway (Harris et al., 2007). However, *T. gondii* infection hinders the activation of the NF-κB signaling pathway in astrocytes (Mason et al., 2004). TIMPs in the CNS can effectively decrease cerebral microvascular permeability and maintain BBB integrity by inhibiting MMP activity (Chen et al., 2013). In the context of *T. gondii* infection, macrophages may penetrate the BBB by promoting the degradation of fibronectin, laminin and type IV collagen, thereby acting as a “Trojan horse” (Da Gama et al., 2004). Further, *T. gondii* infection lead to an upregulation of MMP-8 and MMP-10 by T cells and CNS resident astrocytes. Therefore, to prevent the migration of *T. gondii* to the immune isolation site and evade host immunity, one strategy is to inhibit MMPs activity by enhancing the expression of TIMPs (Clark et al., 2011).

### 2.4 Disruption of the EC tight junction protein (TJP) by *Toxoplasma gondii*

*Toxoplasma gondii* can interact with TJP occludin in intestinal epithelial cells, and it can also disrupt the integrity of the tight junction proteins (TJPs) in retinal pigment epithelial cells (ARPE-19), resulting in increased intercellular permeability and disruption of barrier function (Nogueira et al., 2016; Weight et al., 2015). Although ECs and their TJs constitute the primary barrier that the parasite must traverse to breach the BBB (Masocha and Kristensson, 2012), there are still limited researches on the effect of *T. gondii* on TJPs within BBB ECs. Deletion of the TJP regulator focal adhesion kinase (FAK), also known as protein tyrosine kinase 2 (PTK2), in ECs increases BBB permeability and accordingly elevates the *T. gondii* load in the brain parenchyma (Olivera et al., 2021). FAK is involved in regulating EC adhesion and FAK complex signaling pathways to maintain BBB integrity (Lee et al., 2010). Interestingly, *T. gondii* infection has been reported to inactivate FAK function, thereby transiently disrupting the stability of intercellular TJPs and promoting their intercellular migration (Ross et al., 2019). *T. gondii* infection also leads to an elevation in mRNA and protein levels of brain complement 1q (C1q), which helps clear *T. gondii* from the CNS, but also results in neurodegeneration by degrading neuron connections and synapse loss (Xiao et al., 2016). Additionally, *T. gondii* infection upregulates complement component 3 (C3), further disrupting TJs in the CNS (Huang et al., 2019). Collectively, these effects promote the traversal of *T. gondii* across the BBB through a paracellular migration pathway.

## 3 *Toxoplasma gondii* infection leads to glial cell activation and neuronal injury

Infection with *T. gondii* triggers immune cell infiltration in the brain and parasitism of cysts within neurons, leading to the continuous activation and proliferation of astrocytes, microglia, and blood-derived macrophages (Atmaca et al., 2014). The secretion of IFN-γ by peripheral immune system T lymphocytes is essential to inhibit the proliferation of *T. gondii*, and IFN-γ is also expressed in CNS microglia and blood-derived macrophages (Suzuki et al., 2005). IFN-γ promotes the activation of a series of immune cells, including activating natural killer cells (NK cells), inducing CD8+ T cells to mature and differentiate into effector cytotoxic T cells, and activating macrophages to improve their phagocytosis of *T. gondii* (Gazzinelli et al., 1992). Additionally, it also resists the invasion of *T. gondii* by activating the oxygen-dependent function of DCs and inhibiting the parasite’s proliferation (Nagineni et al., 1996).

Moreover, rodent models of chronic *T. gondii* infection have demonstrated low levels of brain tissue inflammation, characterized by the activation of microglia and astrocytes (Hermes et al., 2008; Estato et al., 2018). These findings highlight concerns regarding neurodegeneration following *T. gondii* infection: Neuroinflammation, as a protective response to regulate neuronal damage, occurs during exposure to toxic metabolites, infections, autoimmune diseases, and aging (Gendelman, 2002). However, it is typically accompanied by various CNS pathological reactions, such as loss of neuronal synapses, reduced neuronal dendritic spine density, neuronal death, and demyelination of neuronal cells (Streit et al., 2004). *T. gondii* cysts are predominantly located in neurons and their maintenance is largely governed by cellular immunity provided by parasite-resident CNS and infiltrating peripheral immune cells (Melzer et al., 2010; Suzuki et al., 1989). In addition, rodent models of chronic *T. gondii* infection show low levels of brain tissue inflammation, manifested as microglia and astrocyte activation (Hermes et al., 2008; Evans et al., 2014). These findings raise concerns about neurodegeneration of the brain after *T. gondii* infection: should the infection remain unresolved, the neuroinflammatory response may persist for years, potentially leading to long-term neurological damage.

### 3.1 *Toxoplasma gondii* infection activates microglia

**Figure 1:**
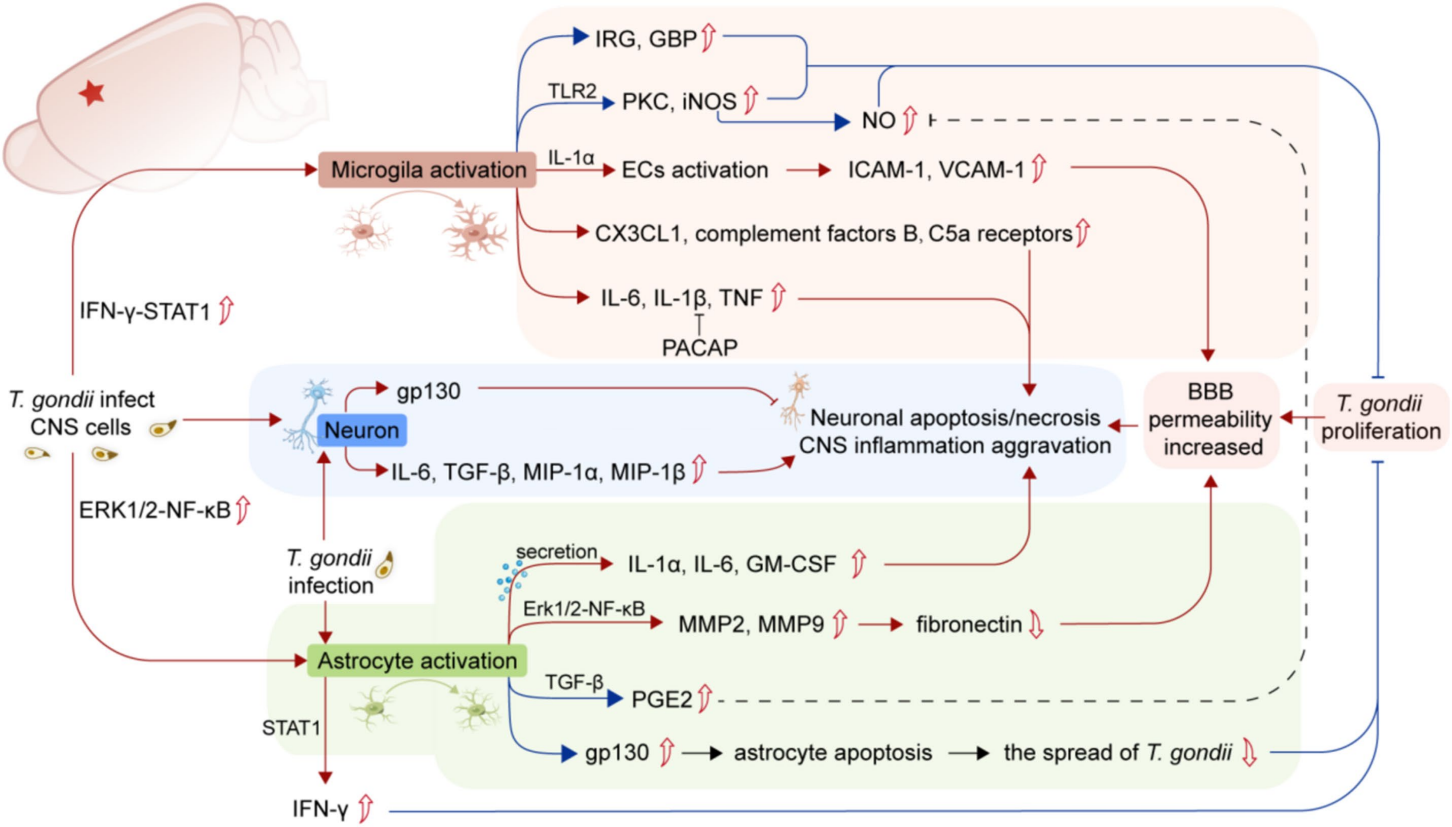
Summary of the mechanisms of the activation of glial cells and neuronal damage caused by *T. gondii* infection. The infection of *T. gondii* in the CNS induces microglia activation and upregulates the expression of pro-inflammatory cytokines, leading to BBB injury and neuronal apoptosis/necrosis (red lines). *T. gondii* infection can also activate astrocytes to produce a wide range of inflammatory factors (red lines), while both microglia and astrocytes play a crucial role in controlling the proliferation of *T. gondii* or reducing neuronal damage (blue lines). The parasitism of *T. gondii* in CNS leads to BBB damage, which exacerbates CNS inflammation. *T. gondii* infection in neurons can also directly lead to neuronal apoptosis/necrosis. CNS, central nervous system; BBB, Blood–brain barrier; IRG, Immune-associated GTase; GBP, Guanylate binding protein; iNOS, Inducible nitric oxide synthase; C5a receptors, Component 5a receptors; ECs, Endothelial cells; ICAM-1, Intercellular adhesion molecule-1; VCAM-1, Vascular adhesion molecule-1; PACAP, Pituitary adenylate cyclase-activating polypeptide; MIP-1α, Macrophage inflammatory proteins-1α; MIP-1β, Macrophage inflammatory proteins-1β; GM-CSF, Granulocyte/macrophage colony-stimulating factor; TGF-β, Transforming growth factor-β; PGE2, Prostaglandin E2; STAT1, Signal transducers and transcriptional activator-1.

*Toxoplasma gondii* can induce the activation of microglia, upregulate the expression of proinflammatory cytokines, and mediate neuronal death in TE (Figure 1). Therefore, inhibiting the activation of microglia may be a new therapeutic strategy for TE (Zhang et al., 2014). Activated microglia cluster at sites of neurodegenerative changes, enveloping or surrounding degenerating neurons, which are the pathogenesis between chronic *T. gondii* infection and neuropsychiatric disorders (Li et al., 2019). After *T. gondii* colonizes the brain, adherence of leucocytes increases, accompanied by increased reactivity of microglia and microglial hypertrophy (Estato et al., 2018). Previous studies have shown that chronic *T. gondii* infection leads to the interaction of CX3CL1, complement and microglia, ultimately promoting phagocytosis of microglia and clearance of degenerative neurons in the cerebral cortex (Li et al., 2019).

A recent study suggests that the IFN-γ-STAT1 signaling pathway participates in microglial activation after *T. gondii* invades the CNS to clear the parasite through immune-associated GTase (IRG) and guanylate binding protein (GBP) activity (Cowan et al., 2022). Microglia can also activate ECs in the CNS to release the proinflammatory cytokine IL-1α (Batista et al., 2020), upregulating the expression of ICAM and VCAM in ECs.

Inflammatory damage caused by invading pathogens can trigger the production of proinflammatory mediators, such as Toll-like receptor 2 (TLR-2) expressed by microglia, as well as promote the expression of protein kinase C and inducible nitric oxide synthase (iNOS). These pathways contribute to the phagocytic activity of microglia against pathogens (Domínguez-Punaro Mde et al., 2010). In addition, *T. gondii* infection can also lead to sustained upregulation of complement factors B and C5a receptors in the mouse brain through the activation of microglia (Shinjyo et al., 2020). However, studies have also shown that the invasion of IFN-γ-activated microglia by *T. gondii* can inhibit the production of iNOS and transforming growth factor-β1 (TGF-β1) and in turn inhibit the proinflammatory effects of activated microglia and prevent neurodegeneration (Rozenfeld et al., 2005).

### 3.2 *Toxoplasma gondii* infection activates astrocytes

Astrocytes are the most abundant glial cells in the CNS, play a crucial role on the growth, development and functional maintenance of neurons as neurotrophic factors. They participate in the release of neurotransmitters to maintain the survival and communication of neurons. Additionally, they are also an important part of the BBB that regulates the permeability of the microvasculature in the CNS (Abbott et al., 2006).

Although *T. gondii* can infect multiple types of cells within the CNS, including astrocytes, microglia, vascular ECs and neurons, in the early stages of tachyzoite invasion, astrocytes show a higher proportion of infection (Gonzalez et al., 2007). After infection with *T. gondii*, the astrocyte-specific marker glial fibrillary acidic protein (GFAP) level, is upregulated, showing widespread astrocyte activation (Nasuhidehnavi and Yap, 2021). Activated astrocytes promote the production of cytokines (including IFN-γ and chemokines), presentation of antigens, and expression of costimulatory molecules (Wilson and Hunter, 2004). Activation of astrocytes caused by *T. gondii* infection is one of the hallmarks of TE, and activated astrocytes in turn can inhibit the replication of *T. gondii* within the brain (Halonen et al., 1998).

Activation of astrocytes by *T. gondii* triggers the release of IL-1α, IL-6, and granulocyte/macrophage colony-stimulating factor (GM-CSF), driving local anti-toxoplasma inflammatory responses in the brain (Fischer et al., 1997). *In vivo* and *in vitro* experiments demonstrate that the ability of IFN-γ to inhibit *T. gondii* replication in astrocytes is dependent on signal transducers and transcriptional activator 1 (STAT1), this inhibition is not observed in mice lacking STAT1 specifically in astrocytes (Hidano et al., 2016). In addition, the soluble factor prostaglandin E2 (PGE2), released by infected astrocytes, can inhibit the production of nitric oxide by IFN-γ-activated microglia, thereby preventing neuronal degeneration (Rozenfeld et al., 2003).

The expression and release of cytokines and chemokines from astrocytes depends on the involvement of Toll-like receptors (TLRs) and specific TLR-binding ligands (Krasowska-Zoladek et al., 2007). TLR is a germline-encoded pattern recognition receptor (PRR) essential for host cell recognition of intracellular parasites, as well as microbial pathogens (Kawai and Akira, 2011). Studies have shown that TLR is involved in interactions between glial cells and neurons, as well as between immune cells and innate cells of the CNS (Kawai and Akira, 2010). TLR-11, a toxoplasma-specific receptor, plays a central role in immune recognition against *T. gondii* infection and is expressed in astrocytes, neurons, and microglia/blood-derived macrophages in the brains of infected mice (Atmaca et al., 2014). Human TLR-5, evolutionarily similar to mouse TLR-11, mediates *T. gondii* actin-binding protein-induced proinflammatory responses in human monocytes (Salazar Gonzalez et al., 2014). TLR-9 mediates distinct inflammatory changes in intestinal and extraintestinal compartments, including the brain (Bereswill et al., 2014). TLR-2 also responds to *T. gondii* infection by participating in the production of CCL2 and TNF. Astrocytes TLR-2 and TLR-4 are involved in the expression and release of IL-1, IL-6, TNF, C-C, or C-X-C chemokines (Bsibsi et al., 2007). In contrast, TLR3 in astrocytes induces the expression of neuroprotective factors and anti-inflammatory cytokines (Liu et al., 2020). Interestingly, treatment with an astrocyte TLR-9 antagonist, oligodeoxynucleotide 2088 (ODN 2088), increased the release of CCL1, thereby promoting the chemotaxis of pericardial macrophages in astroglia-macrophage coculture (Li et al., 2020). Additionally, this antagonist can alter the signaling between astrocytes and macrophages, promoting the polarization of peripheral macrophages toward an M2 phenotype (Li et al., 2020). Moreover, neuron-expressed TLR induces the expression of proinflammatory factors such as IFN-γ and cytokines and can participate in neuronal cell death through apoptosis (Adhikarla et al., 2021). However, the astrocyte TGF-β signaling pathway limits inflammatory responses and reduces neuronal damage during *T. gondii* infection in the CNS (Cekanaviciute et al., 2014). In addition to cytokines and chemokines, MMP-2 and MMP-9 produced by SVG p12 astrocytes line degrade fibronectin, leading to degradation of the BBB extracellular matrix (Lu and Lai, 2013a). NF-κB is involved in regulating the expression of MMP-2 and MMP-9 in *T. gondii-*infected astrocytes, so inhibiting the Erk1/2-NF-κB signaling pathway in astrocytes may represent a potential approach for controlling the development of inflammation in TE (Lu and Lai, 2013b). Additionally, the expression of gp130, a signaling subunit shared by IL-6 family receptors, has been reported to inhibit apoptosis in *T. gondii-*infected astrocytes in mice, which helps control the spread of tachyzoites in the brain (Drögemüller et al., 2008). In summary, invasion of the CNS by *T. gondii* activates host astrocytes to produce various inflammatory factors, which promote the recruitment of macrophages and lymphocytes to the brain and act as antigen-presenting cells expressing costimulatory molecules (Figure 1).

### 3.3 *Toxoplasma gondii* infection leads to neuronal damage

Neurons are the main target cells infected by *T. gondii* within the CNS (Cabral et al., 2016). In contrast to microglia and astrocytes, the parasite can establish persistent infections in neurons, which may be due to the parasite’s directly regulation on neurons. As the infection progresses, *Toxoplasma* cysts in neurons enlarge, their walls thicken, and they assume a rounder shape, all maintaining the integrity of the neuronal cell membrane and concurrently inhibiting neuronal activity (Haroon et al., 2012). Notably, during the chronic phase of *T. gondii* infection in the CNS, the rupture of these cyst walls is a frequent occurrence, allowing bradyzoites to escape and subsequently infect adjacent components of the neuronal vascular unit (NVU) (Hermes et al., 2008).

Studies have shown that *T. gondii* infection in neurons can induce the production of IL-6, TGF-β1, MIP-1α, MIP-1β and other neuronal cytokines and chemokines (Figure 1), which are involved in the immune response against *T. gondii* in the brain immune defenses (Schlüter et al., 2001). Similar to astrocytes, the expression of neuronal gp130 is critical for preventing neuronal loss, excessive inflammation, and lethal processes in TE in mice (Händel et al., 2012). A recent discovery has highlighted that exogenous neuropeptide pituitary adenylate cyclase-activating polypeptide (PACAP) can inhibit the expression of proinflammatory cytokines such as IFN-γ, IL-6, iNOS and IL-1β and alleviate neuronal injury by increasing the expression of brain-derived neurotrophic factor (BDNF) (Figueiredo et al., 2022).

## 4 The co-relation of *Toxoplasma gondii* infection with neurodegenerative and neuropsychiatric disorders

The activation of the immune system in the CNS is a common denominator in neuropsychiatric disorders such as schizophrenia and neurodegenerative disorders such as Alzheimer’s disease (AD) (Muzio et al., 2021). Researches have linked *T. gondii* infection to an increased risk of these brain disorders, such as schizophrenia (Torrey et al., 2012), AD (Kusbeci et al., 2011), and depression (Hsu et al., 2014), by inducing a systemic immune response that may contribute to disease pathology in the host. *T. gondii* seropositivity in the elderly is associated with a decline in perceptive and cognitive capabilities (Nimgaonkar et al., 2016). In mammalian studies, *T. gondii* infection has been shown to cause neophobia in animals and affect learning, memory and movement (Kaushik et al., 2012). Depression-like behavior in mice can occur during *T. gondii* acute infection, characterized by anhedonia and despair-like behavior (Mahmoud et al., 2017). Neuroinflammation and cytokine imbalance induced by *T. gondii* during chronic infection promote the progression of neuropsychiatric diseases by altering neurotransmitter metabolism, tryptophan metabolism, host immune function and systemic hormone levels (Elsheikha et al., 2016).

Analysis of mice with chronic *T. gondii* infection revealed that the density and continuity of nerve fibers were damaged (Parlog et al., 2014). In the brains of *T. gondii*-infected mice, the morphogenesis of neurons changed, such as the reduction of dendritic spines and the blocking of neural network activities (David et al., 2016). Population studies found that compared with *T. gondii*-negative patients, *T. gondii*-positive schizophrenic patients had significantly reduced cortical gray matter volume (Horacek et al., 2012).

### 4.1 *Toxoplasma gondii* infection and Alzheimer’s disease

Alzheimer’s disease (AD) is a prevalent degenerative disease of the CNS characterized by progressive memory loss, cognitive decline, and various neuropsychiatric symptoms and behavioral disorders (Nayeri et al., 2021). It is well known that neuroinflammation-induced neuronal degeneration plays a key role in the pathogenesis of chronic neurodegenerative diseases. A large number of studies have shown that there are many inflammatory markers in the brains of AD patients, including an increase in inflammatory cytokines and chemokines and the accumulation of activated microglia in damaged areas (Torres et al., 2018).

**Figure 2:**
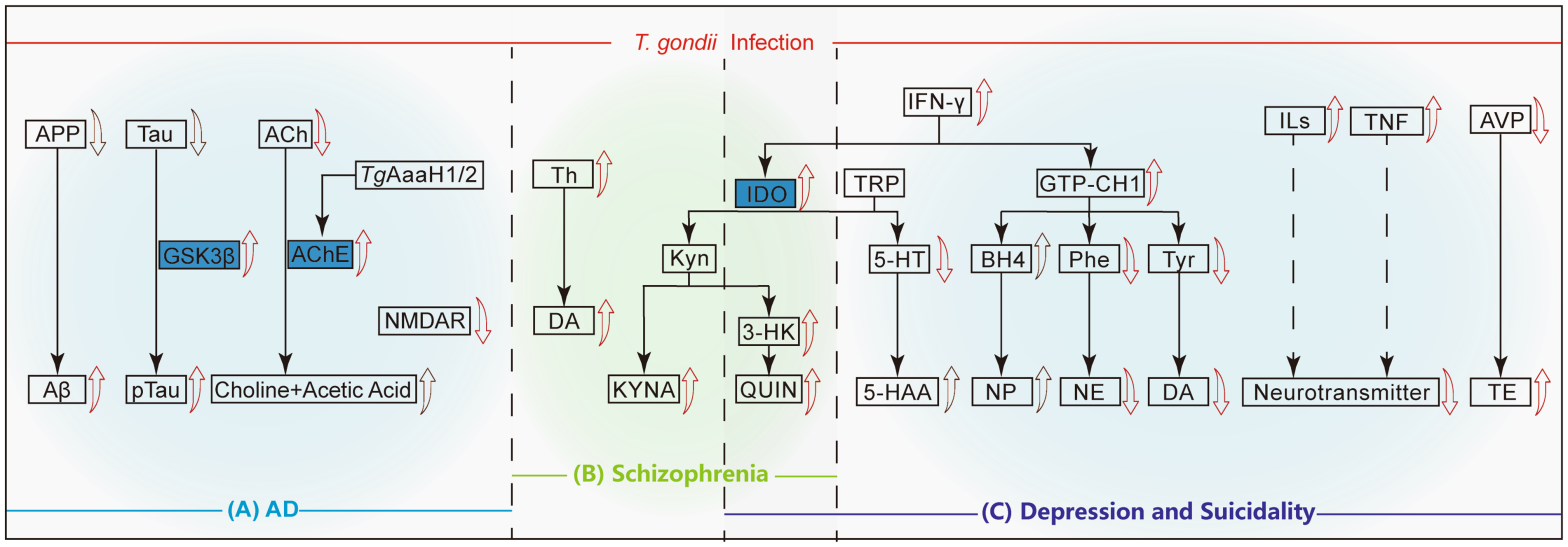
Mechanisms of neuropsychiatric disorders causing by *T. gondii* infection. The red arrows represent that *T. gondii* infection directly leads to the up regulation/activation or degradation of this protein/compound, and the black arrows indicates a decrease in the upstream proteins or an accumulation of metabolites due to the promotion of the synthesis pathways. The solid lines mean the direct metabolic pathways, while the dashed lines indicates that other substances may be involved in this pathway. **(A)** In Alzheimer’s disease, *T. gondii* infection results in excessive conversion of APP to Aβ and Tau to p-Tau, accompanied by the downregulation of ACh and NMDAR. **(B)** In Schizophrenia, *T. gondii* infection can directly promote the production of Th, DA and activate the KYN pathway downstream of IDO, which is also related to the occurrence of depression. **(C)** In depression and suicidality cases, the up-regulation of IFN-γ leads to the decrease of the inhibitory neurotransmitter 5-HT downstream of TRP. On the other hand, IFN-γ up-regulates GTP-CH1, which ultimately leads to the down-regulation of NE and DA. *T. gondii* infection induces the up-regulation of ILs and TNF, and the down-regulation of AVP, which eventually leads to an imbalance of the neurotransmitter and up-regulation of TE level, respectively. APP, Amyloid precursor protein; Aβ, Beta-amyloid protein; GSK3β, Glycogen synthase kinase-3β; ACh, Acetylcholine; AChE, Acetylcholinesterase; NMDAR, N-methyl-D-aspartate receptor; TH, Tyrosine hydroxylase; DA, Dopamine; IDO, Indoleamine 2,3-dioxygenase; Kyn, Kynurenine; KYNA, Kynurenic acid; 3-HK, 3-hydroxykynurenine; QUIN, Quinolinic acid; TRP, tryptophan; 5-HT, 5-hydroxytryptamine; 5-HAA/5-HIAA, 5-hydroxyindole acetic acid; GTP-CH1, Guanosine triphosphate cyclohydrolase-1; BH4, Tetrahydrobiopterin; NP, Neopterin; Phe, Phenylalanine; NE, Norepinephrine; Tyr, Tyrosine; DA, Dopamine; ILs, Interleukins; TNF, Tumor necrosis factor; AVP, Arginine vasopressin; TE, Testosterone.

The hallmark of AD pathology involves extensive deposits of beta-amyloid protein (Aβ) forming amyloid plaques in the brain, leading to significant neuronal damage (Lee et al., 2010). After *T. gondii* infection, the amyloid precursor protein (APP) in host’s CNS is abnormally cleaved, and its expression level drops, resulting in a large amount of Aβ deposition in brain tissue (Figure 2A). Acetylcholine (ACh), an essential neurotransmitter implicated in AD, when rapidly hydrolyzed by acetylcholinesterase (AChE) into acetate and choline, leads to deficits in learning, memory, and cognition (Ferreira-Vieira et al., 2016). *T. gondii* infection results in high AChE release and lower ACh levels, leading to learning and memory impairment in the infected host (Nayeri et al., 2021). Another important pathological change in AD is the hyperphosphorylation of tau protein, which leads to neurofibrillary tangles, structural changes in neuronal cytoskeletal proteins, and ultimately neuronal death. *T. gondii* infection leads to tau phosphorylation by activating glycogen synthase kinase 3β (GSK3β), leading to significant apoptosis of hippocampal neurons (Tao et al., 2020). In addition, the anxiety-like behavior induced by *T. gondii* infection in mice may be related to reduced N-methyl-D-aspartate receptor (NMDAR) expression and loss of olfactory sensory neurons (Torres et al., 2018).

Interestingly, chronic *T. gondii* infection can improve Aβ deposition in AD by activating the immune system, mainly by recruiting monocytes, enhancing monocytic phagocytosis and promoting soluble Aβ degradation; the excessive activation of glial cells by *T. gondii* may lead to Aβ degradation (Möhle et al., 2016). Replicating this phenomenon in animal models with varying infection levels could deepen our understanding of the relationship between *T. gondii* and brain disorders, presenting new avenues for research into treatments for conditions like AD.

In addition, the specific mechanism of how *T. gondii* infection affects AChE release is still unclear. Previous studies have shown that *T. gondii* infection can increase the levels of AChE in the brains of mice (Tonin et al., 2014), and research on improving AD pathology by targeting *T. gondii* infection may have significant research implications.

### 4.2 *Toxoplasma gondii* infection and schizophrenia

Schizophrenia is a chronic long-term mental disorder characterized by a progressive deterioration in sensory, cognitive, emotional, and behavioral functions (Tandon et al., 2013). Risk factors such as childhood trauma, obstetric complications, drug abuse, and neuroinflammation caused by infection can all contribute to the development of schizophrenia (Radhakrishnan et al., 2017; Stilo and Murray, 2019). At present, most studies use serum anti-*T. gondii* IgG as an indicator of *T. gondii* infection, and the serum positivity rate of *Toxoplasma* IgG in schizophrenia patients is significantly higher than that in the control group (Oncu-Oner and Can, 2022). A meta-analysis showed that the serum positivity rate of *T. gondii* in schizophrenic patients was 2.73 times higher than that in the general population (95% confidence interval, 2.10–3.60; *p* < 0.000001) (Torrey et al., 2007), suggesting a moderate to strong association between *T. gondii* infection and schizophrenia, and raising the possibility that *T. gondii* infection could contribute to the development of this mental disorder (Kezai et al., 2020). Chronic infection with *T. gondii* may cause neuroinflammation, which in turn can lead to neurotransmitter imbalances and subsequent psychopathological symptoms, potentially exacerbating the progression of schizophrenia.

An important factor in schizophrenia is the dysregulation of dopamine (DA), a neurotransmitter whose elevated levels are implicated in the disease (Willner, 1997). There is an association between DA and *T. gondii* infection and schizophrenia, and it is associated with an increase in the hippocampus and other specific regions (Skallová et al., 2006). In *T. gondii* infected mouse models, abnormal elevation in DA concentration in the synaptic cleft of neurons lead to schizophrenia (Yin et al., 2022). The upregulation of tyrosine hydroxylase (TH) expression in the infected host brain may be related to *TgAaaH1* and *TgAaaH2*, which encode TH in the T*oxoplasma* genome, leading to excessive production of DA in infected nerve cells (Gaskell et al., 2009). An increase in DA concentration was also detected in cysts and surrounding tissues in patients with schizophrenia (Flegr, 2015).

The increase in kynurenine (Kyn) metabolites is closely related to the onset of schizophrenia (Zhou et al., 2022). Studies have found that *T. gondii* infection may lead to an increase in 3-hydroxykynurenine (3-HK, metabolites of Kyn), quinolinic acid (QUIN) and kynurenic acid (KYNA) in the brain through activating microglia and astrocytes (Figure 2B). The synthesis of KYNA is initiated by starting the oxidative cycle of tryptophan through indoleamine 2,3-dioxygenase (IDO) and/or tryptophan dioxygenase (TDO). Due to the increased TDO activity in the brains of individuals with genetic susceptibility to schizophrenia, the increase in these Kyn metabolites is exacerbated. The increase in KYNA levels in turn leads to excessive inhibition of glutamatergic and cholinergic neurotransmission, which can contribute to the onset of schizophrenia (Schwarcz and Hunter, 2007; Notarangelo et al., 2014).

The associations outlined above offer compelling avenues for further research into the role of *T. gondii* infection in the etiology and pathophysiology of schizophrenia. Understanding these complex interactions could potentially lead to the development of new therapeutic interventions targeting these specific mechanisms.

### 4.3 *Toxoplasma gondii* infection and depression and suicidal tendencies

Depression is a common mental disorder characterized by significant and persistent low mood, slow thinking, cognitive impairment and reduced volitional activity (Xu et al., 2020). Depression is closely related to suicidal tendencies, and suicidal behavior is the most common complication of depression (Conejero et al., 2018). *T. gondii* infection has been reported to be associated with depression and suicidal tendencies (Okusaga et al., 2011), and chonic *T. gondii* infection may be a risk factor for depression and suicidal behavior (Kamal et al., 2022). After *T. gondii* infection, the host brain releases inflammatory cytokines, leading to neuroendocrine and immune system dysfunction and increasing the risk of depression in the host by exacerbating anxiety and depressive-like behavior (Bay-Richter et al., 2019).

Serotonin (also called 5-hydroxytryptamine, 5-HT) is a neurotransmitter with high concentration in the cerebral cortex and nerve synapses, and it is an important substance regulated to nerve activity. *T. gondii* infection leads to IFN-γ production and brain IDO activation, which degrades tryptophan and causes a decrease in 5-HT synthesis, ultimately inducing depression and suicidal behavior (Ihara et al., 2016; Mahmoud et al., 2016). *T. gondii* bradyzoites reactivation promotes the conversion of 5-HT to its main metabolite, 5-hydroxyindoleacetic acid (Mahmoud et al., 2016). Therefore, the enhancement of the tryptophan catabolic shunt and serotonin conversion may be associated with the development of depression-like behavior in *T. gondii*-reactivated mice, which may be triggered by immune imbalance (Mahmoud et al., 2016). In addition, the increase in Kyn production mediated by IDO through the kynurenine pathway may induce core symptoms of depression, such as anhedonia, or further transform into downstream neuroreactive metabolites such as KYNA and QUIN. Increases in these metabolites can be detected in the serum of patients with depression (Mahmoud et al., 2016).

After *T. gondii* infection, IFN-γ and other proinflammatory cytokines induce the activation of guanosine triphosphate cyclohydrolase-1 (GTP-CH1), which reduces the levels of phenylalanine (Phe, a precursor to norepinephrine) and tyrosine (Tyr, a precursor to dopamine), and may lead to a decrease in the levels of norepinephrine (NE) and DA in the synaptic cleft (Figure 2C), subsequently resulting in the onset of depression (Hsu et al., 2014; Kamal et al., 2022). The levels of inflammatory cytokines in the serum of patients with depression, including interleukins and TNF, are significantly elevated, and may indirectly reduce the levels of neurotransmitters in the brain (Catena-Dell'Osso et al., 2011). In addition, research has shown that after infection, the promoter of the arginine vasopressin (AVP) gene in the host is hypomethylated, leading to increased AVP expression, increased testosterone secretion (Figure 2C), and ultimately reduces fear of cat urine odor in infected rats, which may be linked to the exhibition of suicidal behaviors (Hari Dass and Vyas, 2014).

## 5 Discussion and prospects

Similar to other pathogens that can invade the CNS, the pathogenesis of various neuropsychiatric disorders following *T. gondii* infection is attributed to the proinflammatory immune response and neurotransmitter dysregulation mediated by the secretion and subsequent activation of systemic inflammatory cytokines within the CNS (McConkey et al., 2013).

Neurons, astrocytes, microglia and ECs of the CNS all contribute to the disruption of the brain microenvironment through an inflammatory network that includes cytokines, chemokines, adhesion molecules, integrins and complements (Gilhus and Deuschl, 2019). Infected astrocytes and microglia can eliminate the parasite via lysosomal degradation, but neurons lack this pathway (Morales et al., 2014). Therefore, this may be the reason why *T. gondii* has a high infection rate in astrocytes, but cysts can only be observed in neurons during chronic infection. The latest study shows that neurons stimulated by IFN-γ can eradicate invading *T. gondii* in an IRG-dependent manner (Chandrasekaran et al., 2022). However, the mechanism by which *T. gondii* infects neurons and maintains cell membrane integrity without inducing neuronal apoptosis remains to be further investigated.

Interestingly, the activation of the host immune system not only leads to the clearance of *T. gondii* but also promotes the invasion of *T. gondii* into the CNS in a “Trojan horse”-like manner. This raises the question: Could moderating the host’s anti-inflammatory response during infection prevent encephalitis and neuropsychiatric disorders? For example, histamine H1 receptor antagonists, known as anti-allergic drugs, have been reported to alleviate cytokine storms in COVID-19 patients (Qu et al., 2021). This raises the question of whether such drugs could also temper excessive immune activation against *T. gondii*.

Unfortunately, research on modulating the host’s immune response or managing inflammation after *T. gondii* infection is still limited. Therefore, it may be beneficial to adapt immune signaling pathways knowledge from the study of other pathogenic infections to *T. gondii* treatment. Studies have shown that the intracellular signaling pathways leading to the activation of glial cells during infections, including those induced by *T. gondii*, involve TLR activation, signal transduction through apoptotic receptors, NF-κB signaling pathway activation, and increased TNF and IL-1β secretion (Figure 1). Consequently, treatments for CNS inflammation resulting from *T. gondii* could potentially benefit from strategies used against other infectious agents.

The ability of *T. gondii* infection to activate cell signaling pathways similar to those triggered by other pathogens, is primarily due to the secretion of IFN-γ by host cells upon pathogen recognition. This secretion, in turn, activates a cascade of anti-infective immune responses downstream of IFN-γ, functioning both intra-and intercellularly. *Toxoplasma gondii* secretes diverse antigens into the host’s parasitophorous vacuoles (PV) before secretion of IFN-γ, during its invasion and proliferation, potentially leading to the activation of specific host cell signaling pathways. Furthermore, *T. gondii* alters the function of host cells by secreting various effector molecules, including rhoptry proteins (ROPs) into the cytoplasm during invasion, and dense granule antigens (GRAs) targeting the parasitophorous vacuole membrane (PVM), cytoplasm or nucleus during proliferation (Rosenberg and Sibley, 2021).

ROPs such as ROP16, ROP17, ROP18 and ROP5, are secreted into the host cytoplasm by *T. gondii,* typically at the onset of host cell invasion. Once inside the parasitophorous vacuole (PV), *T. gondii* secretes GRAs targeting the PVM (e.g., GRA15 and MAF1), host cytoplasm (e.g., GRA18), or host nucleus (e.g., GRA16, GRA18 and GRA24) (Wang et al., 2020). The GRA proteins mainly inhibits the host immune response and promotes parasite replication (Hunter and Sibley, 2012), with GRA15 also capable of suppressing the IFN-γ-induced antiparasitic response in human neurons (Bando et al., 2019). In addition, some effector proteins secreted by *T. gondii* may interfere with the host immune response (Hakimi et al., 2017), potentially leading to the death of neurons and glial cells in the host brain, thus impairing normal nervous system function. Unfortunately, research into the secretory proteins of *T. gondii* and their role in host CNS inflammation remains limited. Identifying the specific signaling pathways targeted by *T. gondii* effector molecules could be instrumental in devising targeted interventions to neutralize virulent parasite strains and mitigate excessive inflammatory responses in the CNS.

The formation of *T. gondii* cysts in the host involves the transformation of tachyzoites into bradyzoites and the subsequent formation of cyst walls. The main component of the cyst wall is *α*-linked N-acetylgalactosamine, which is elastic and helps the cysts evade the host’s immune recognition. The persistent inflammation caused by this space-occupying lesion is not only an important factor in the development of neurological and psychiatric diseases but also a “time bomb” for immunocompromised hosts, such as advanced cancer patients and organ transplant recipients. Currently, multiple genes have been identified that regulate the interconversion between the bradyzoite and tachyzoite of *T. gondii* (Waldman et al., 2020). However, critical evidence is still lacking regarding how *T. gondii* cysts respond to a compromised host immune system. Recent studies indicate that bradyzoites within cysts may sense the host’s immune status through the regulation of transcription factors such as AP2 (Kim, 2018). As a result, *T. gondii* adjusts its growth and reproduction strategy, reverting to tachyzoites for rapid multiplication when conditions are favorable (Arranz-Solís and Saeij, 2022). Therefore, studies of this conversion mechanism are of great significance for advancing our understanding of *T. gondii* infections and improving treatment strategies.

## Glossary

3-HK: 3-hydroxykynurenine
5-HT: 5-hydroxytryptamine
ACh: Acetylcholine
AChE: Acetylcholinesterase
AD: Alzheimer’s disease
APP: Amyloid precursor protein
AVP: Arginine vasopressin
Aβ: Beta-amyloid protein
BBB: Blood–brain barrier
BDNF: Brain-derived neurotrophic factor
C1q: Complement 1q
C3: Component 3
CNS: Central nervous system
DA: Dopamine
DCs: Dendritic cells
ECM: Extracellular matrix
ECs: Endothelial cells
GBP: Guanylate binding protein
GFAP: Glial fibrillary acidic protein
GM-CSF: Granulocyte/macrophage colony-stimulating factor
GRAs: Granule antigens
GROα: Growth-related carcinogens α
GSK3β: Glycogen synthase kinase-3β
GTP-CH1: Guanosine triphosphate cyclohydrolase-1
Hela: Human cervical carcinoma epithelial cells
HFF: Human preputial fibroblasts
ICAM-1: Intercellular adhesion molecule-1
IDO: Indoleamine 2,3-dioxygenase
iNOS: Inducible nitric oxide synthase
IRG: Immune-associated GTase
Kyn: Kynurenine
KYNA: Kynurenic acid
MIP-1α: Macrophage inflammatory proteins-1α
MIP-1β: Macrophage inflammatory proteins-1β
MT1-MMP: Membrane type 1 matrix metalloproteinase
NK cells: Natural killer cells
NMDAR: N-methyl-D-aspartate receptor
NVU: Neuronal vascular unit
ODN: Oligodeoxynucleotide
PACAP: Pituitary adenylate cyclase-activating polypeptide
PGE2: Prostaglandin E2
Phe: Phenylalanine
PRR: Pattern recognition receptor
PV: Parasitophorous vacuoles
PVM: Parasitophorous vacuole membrane
QUIN: Quinolinic acid
ROPs: Rhoptry proteins
STAT1: Signal transducers and transcriptional activator-1
TDO: Tryptophan dioxygenase
TE: Toxoplasma encephalitis
TEM: Trans-endothelial migrate
TGF-β1: Transforming growth factor-β1
TH: Tyrosine hydroxylase
TIMPs: Tissue metalloproteinase inhibitors
TJ: Tight junctions
TJP: Tight junction protein
TLR: Toll-like receptor
Tyr: Tyrosine
VCAM-1: Vascular adhesion molecule-1
